# *PAX3* gene deletion detected by microarray analysis in a girl with hearing loss

**DOI:** 10.1186/1755-8166-7-30

**Published:** 2014-04-29

**Authors:** Malgorzata Drozniewska, Olga Haus

**Affiliations:** 1 Department of Clinical Genetics, Collegium Medicum Nicolaus Copernicus University, Skłodowskiej-Curie 9, 85-094 Bydgoszcz, Poland; 2 West Midlands Genetics Laboratories, Birmingham Women's Hospital NHS Foundation Trust, Edgbaston, B15 2TG Birmingham, UK; 3 Department of Hematology, Blood Cancer and Bone Marrow Transplantation, Medical University, Pasteura 4, 50-367 Wroclaw, Poland

**Keywords:** *PAX3* gene, Array-CGH, Hearing loss, Waardenburg syndrome, Craniofacial-deafness-hand syndrome

## Abstract

Deletions of the *PAX3* gene have been rarely reported in the literature. Mutations of this gene are a common cause of Waardenburg syndrome type 1 and 3. We report a 16 year old female presenting hearing loss and normal intellectual development, without major features of Waardenburg syndrome type 1, and without family history of the syndrome. Her phenotype, however, overlaps with features of craniofacial-deafness-hand syndrome. Microarray analysis showed ~862 kb *de novo* deletion at 2q36.1 including *PAX3*. The above findings suggest that the rearrangement found in our patient appeared *de novo* and with high probability is a cause of her phenotype.

## Background

Hearing loss is a common feature which can be present as an isolated form or be one of an auditory phenotype symptoms [1]. Inherited hearing loss can be transmitted in an autosomal dominant, autosomal recessive, X-linked or even in mitochondrial mode of inheritance. Although most of hearing loss cases are nonsyndromic, accompanying abnormalities can be present.

Hearing loss can be present in more than 400 genetic syndromes. Waardenburg syndrome (WS) is the most common type of hearing loss inherited as autosomal dominant trait. Phenotype associated with this syndrome includes, apart from hearing loss of various degree, pigmentary abnormalities of the skin, eye (heterochromia irides or bright blue irides) and hair (white forelock). Four types of WS have been distinguished, depending on the presence of other abnormalities. Patients with WS type 1 (WS1) present mutation within *Paired Box 3* (*PAX3*) gene, however it has been shown to be present in only 45% of the WS1 syndrome. Heterozygous mutations of this gene have been reported in both sporadic and familial cases [1]. Up to date more than 100 *PAX3* mutations have been recorded in the Human Gene Mutations Database, of which about 50% are missense/nonsense mutations. Partial or whole gene deletions have been reported in ~10% of patients [2,3]. Rare subtype, craniofacial-deafness-hand syndrome (CDHS), can also be caused by *PAX3* mutations.

*PAX3* gene belongs to the transcription factors paired box family and has been mapped to chromosome 2q35. As shown by Bondurand et al., *PAX3*, together with *SOX10*, strongly activates microphtalmia-associated transcription factor (MITF) and contributes to pigmentation abnormalities [4].

We report on a female patient with congenital sensorineural hearing loss with minor facial dysmorphism partially overlapping with features characteristic for Waardenburg syndrome 1 and craniofacial-deafness-hand syndrome in which *PAX3de novo* deletion has been identified by microarray analysis.

## Case presentation

### Clinical report

The 16 years old girl was referred for genetic counselling due to congenital hearing loss and subtle dysmorpic features. The proband is the second child of non-consanguineous parents. She has two healthy sisters. She was born at term after an uneventful pregnancy. Her birth weight was 3200 g, length 53 cm. Apgar score was 10. Synophrys and low set ears were noted at birth.

Her early psychomotor development was normal, without any notable delay. Later she presented speech impairment due to hearing problems, noted at the age of 2. She attended a school for children with hearing impairment.

At the age of 16 she was hospitalised at the Endocrinology Unit due to hirsutism and hypercholesterolemia. Physical examination revealed seborrheic dermatitis, astigmatism, and profound sensorineural hearing loss. Hirsutism was scored at 6 according to the Ferriman-Gallwey scale. Sex hormone levels were normal.

**Figure 1:**
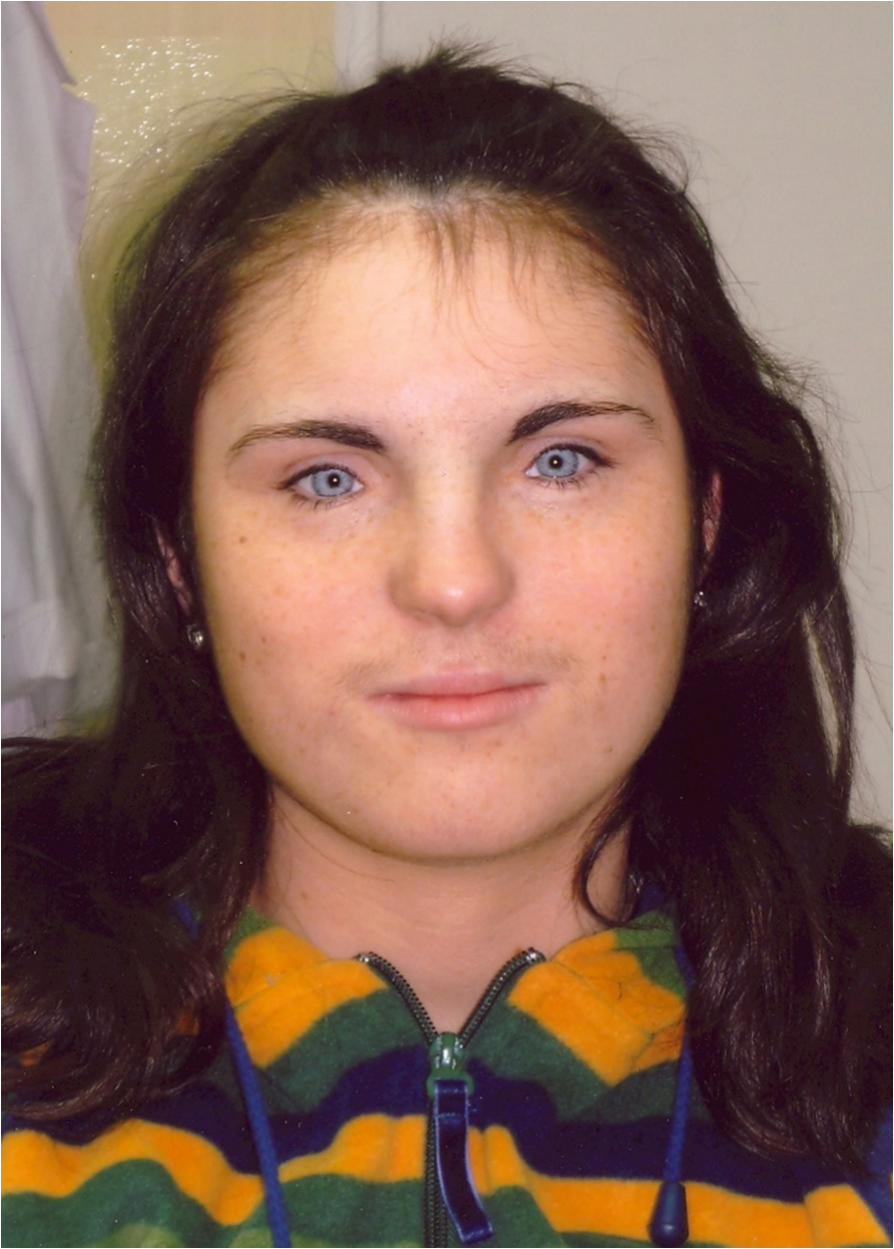
**Facial appearance of the patient at the age of 16.** Note brilliant blue irides, hypertelorism, dystopia canthorum, hirsutism.

Phenotype examination at the genetic counselling centre revealed the presence of wide set eyes of brilliant blue irides, dystopia canthorum, shortened upslanting palpebral fissures, hypoplastic alae nasi, hirsutism (Figure 1). Please note that synophrys is not visible as she keeps her eyebrows plucked. She did not present white forelock characteristic for WS, however it cannot be excluded that she dyed her hair (despite denying it). Intellectual development was normal. Both parents were phenotypically normal and neither of them presented any features suggestive of WS.

### Methods of detection

#### Cytogenetics

Chromosomal analysis was performed according to standard procedures on GTG-banded metaphase spreads, obtained from peripheral blood lymphocytes.

#### Array-CGH

An oligo array-CGH was performed using the Human Genome CGH Microarray Kit and SurePrint G3 4x180K Human Kit (Agilent Technologies, Santa Clara, CA, USA), according to the manufacturer’s protocols. The Agilent Feature Extraction software has been used to perform image analysis. Array data were compared with the human genome reference sequence hg19 (February 2009). Genomic DNA was extracted from peripheral blood lymphocytes.

#### Method of confirmation (FISH)

Fluorescence *in situ* hybridisation (FISH) was performed on metaphase spreads by using *PAX3* Breakapart Probe (Cytocell, Cambridge, UK) according to manufacturer’s protocol. Metaphases were analysed with Nikon Eclipse fluorescent microscope. Images were analysed and archived with Applied Spectral Imaging software (Applied Spectral Imaging, Edingen, Neckerhausen, Germany).

## Results

Cytogenetic GTG analysis revealed normal female karyotype.

**Figure 2:**
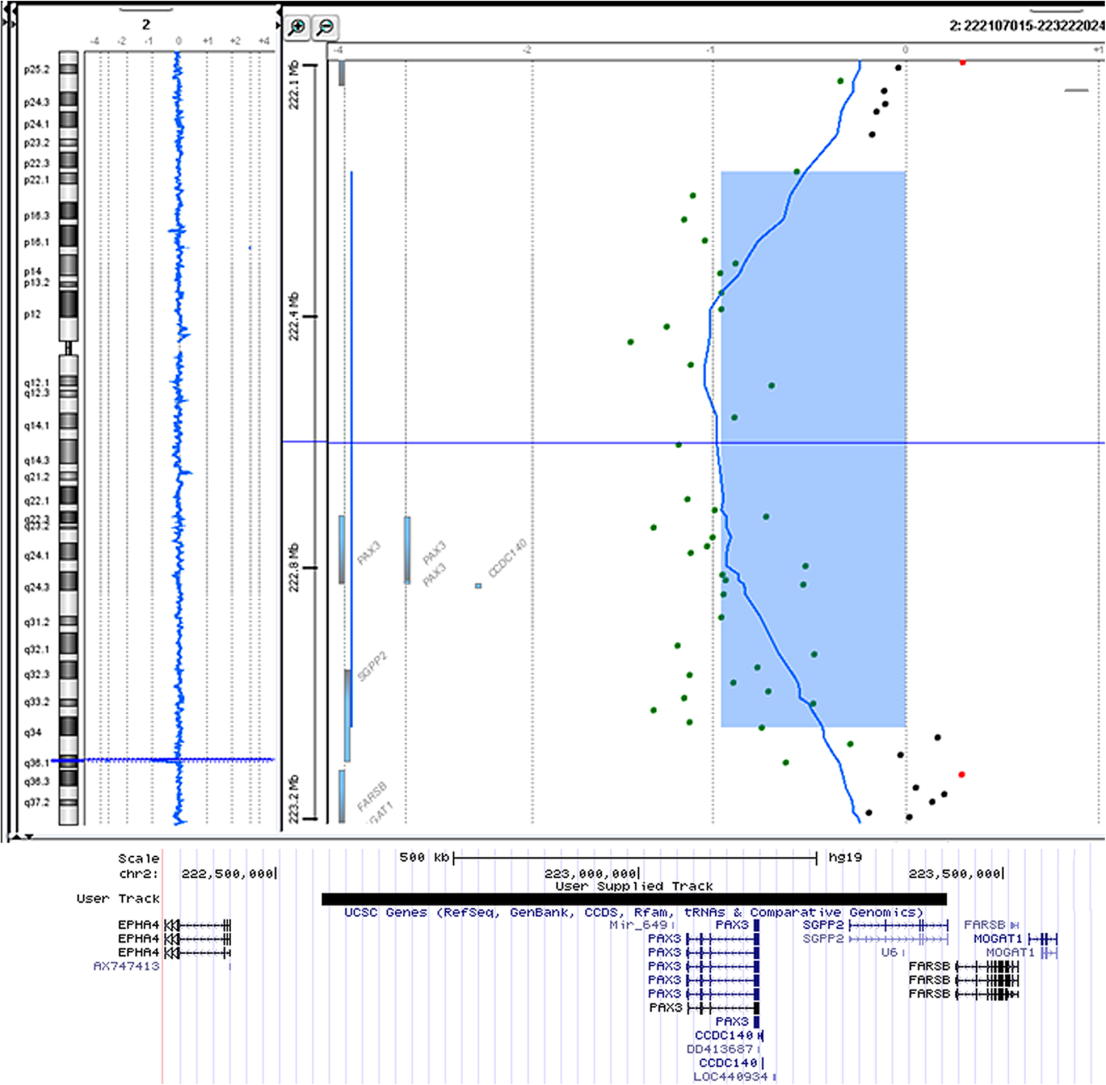
**Array-CGH profile using Agilent 180 K microarray showing deletion of 2q36.1 including *PAX3 *gene.** Below – overview of the deletion region.

Array CGH experiment disclosed an interstitial deletion within long arm of chromosome 2. The deletion region was found to be ~862 kb in size and ranged between oligos 222,562,885-223,424,791 (UCSC Genome Browser on Human, Feb. 2009 (GRCh37/hg19) Assembly). This region is localised within band 2q36.1 and contains *PAX3* gene, *CCSC140* gene and a part of *SGPP2*. Overview of the deleted region, together with its aCGH profile is shown on Figure 2.

FISH examination using *PAX3* break apart probe confirmed the aCGH finding.

**Figure 3:**
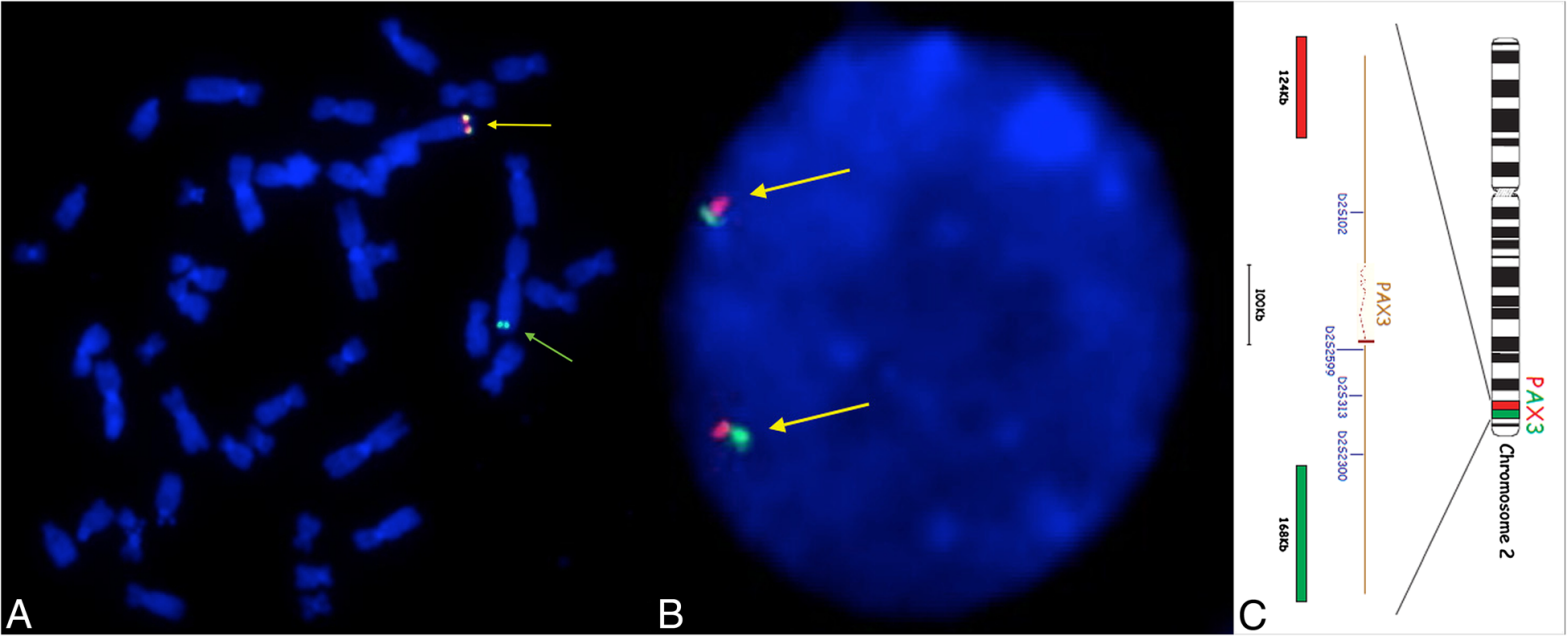
**FISH results with *PAX3 *breakapart probe (Auqarius, Cytocell). A** – patient’s metaphase spread. Arrows indicate fluorescent signals. Yellow arrow shows normal chromosome 2 with both fluorescent signals present. Note absence of one red signal on one of the chromosomes 2, indicated by green arrow. **B** – patient’s mother blood cell nucleus. Yellow arrows indicate fluorescent signals showing no deletion within examined loci. The same signal pattern was observed in patient’s father. **C** – schematic overview of *PAX3* probe (Vysis). Red bar represents proximal region of probe coverage (observed as red signal), green bar – distal region (observed as green signal).

Combined parental follow-up by standard cytogenetics and microarray testing showed no evidence of deletion of 2q36.1 in either parent. FISH studies using the same probe excluded deletion or any balanced rearrangement involving 2q36.1 region. Figure 3 shows FISH results of the proband (3A) and proband’s mother (3B).

Molecular investigation of *GJB2* gene in the girl excluded the presence of its most common mutation, del35G.

The above findings suggest that the rearrangement found in our patient appeared *de novo* and with high probability is a cause of her phenotype.

## Discussion

We report a patient with a *de novo* 2q36.1 deletion of 862 kb, including *PAX3* gene, which is a member of the transcription factors family [4-6]. In humans, constitutional mutations of *PAX3* lead to Waardenburg syndrome (WS) or Craniofacial-deafness-hand (CFDS) syndrome [1,7]. Acquired mutations or rearrangements of *PAX3* may cause alveolar rhabdomyosarcoma [5]. *PAX3* gene contains 10 exons and encodes protein of 98% homology to the mouse orthologue [3,5].

Craniofacial-deafness-hand syndrome (MIM #122880) was first described by Sommer et al. in 1983 [7]. Several follow-up studies were performed since, and genotype-phenotype correlation was established.

Point mutations of *PAX3* account for ~90% of WS1 and WS3 (Waardenburg Syndrome type 3) in patients meeting clinical diagnostic criteria, whereas partial and whole *PAX3* deletions can be causal in only ~6% of cases [8]. Diagnostic tests are based mainly on sequencing of genes involved in WS etiology, however MLPA technique can also be of use [8,9].

**Figure 4:**
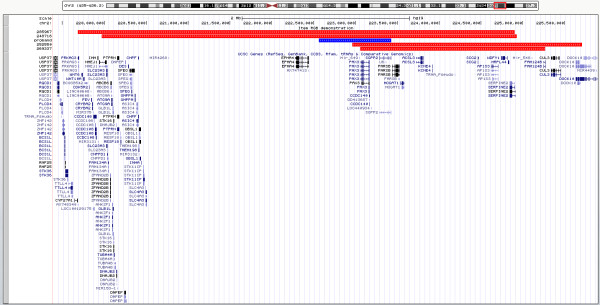
**Schematic comparison of 2q deletions including *PAX3 *recorded in the Decipher database and presented case.** Region of deletion seen in our patient is represented by blue bar. Red bars represent Decipher cases. ID numbers are shown on sidebar.

There are larger deletions of chromosome 2q recorded in the Decipher database, which also include *PAX3* gene. Of these cases one shows similar phenotype to our patient, including synophrys, hypertelorism and iris pigmentation disturbances (ID: 248718). Deletion reported in this patient is 4.95 Mb in size, and its size likely contributes to other clinical features, such as intellectual disability. Comparison of cases recorded in Decipher database and this case is shown on Figure 4.

Previous reports suggested that *PAX3* deletions did not necessarily result in distinct WS phenotype, which can raise the question of *PAX3* penetrance and associated variable phenotype. In addition authors suggested that size of rearrangement can also affect manifestation of WS clinical features [8], however no relationship was found between severity of clinical features and the type of mutation [3].

It is not possible to make a strict correlation between our patient’s phenotype and either WS or CFDS. The diagnosis of Waardenburg syndrome can be made when at least two major or one major and two minor phenotypic criteria are met. Presence of dystopia canthorum and hearing loss (major criteria) together with synophrys, hypoplastic alae nasi and bright blue irides (minor criteria) could be convincing enough to make a diagnosis of WS1. However, comparison of the phenotypes of the two syndromes suggests that the patient could also be qualified as having craniofacial-deafness-hand syndrome. Despite different type of genomic changes described in our patient and patients reported in the literature, similar dysmorphic features and normal intellectual development can lead to conclusion that these patients can be classified into the same group. Comparison of clinical features present in described case and in patients reported by Sommer and Bartholomew [7] Gad et al. [1] is shown in Table 1.

**Table 1:** Review of clinical features present in our patient and in patients with CDHS or variant WS1 reported in the literature

**Phenotype**	**Present case**	**Sommer and Bartholomew **[7]	**Gad et al. **[1]
Flat facial profile	-	+	+
Hypertelorism	+	+	+
Downslanting palpebral fissures	+	+	+
Heterochromia of irides	-	-	-
Synophrys	+	-	-
Depressed nasal bridge	-	+	+
Contractures of digits	-	+	+
Hirsutism	+	-	-
Normal intelligence	+	+	+
Hearing loss	+	+	+
Pigmentation abnormalities	-	-	-
Dystopia canthorum	+	-	-
Brilliant blue irides	+	-	-

Legend: (+) presence of feature, (−) absence of feature.

Further molecular analysis of the second allele is to be considered in terms of any mutations present.

## Conclusion

In conclusion, our case can be another example of *PAX3* rearrangement causing distinct but variable phenotype. It also shows that it may be recommended to perform array-CGH analysis in addition to *PAX3* mutations analysis in the patients with hearing loss and dysmorphy.

## Consent

Approval to conduct the study was granted by the local Bioethics Committee. Written informed consent was obtained from parents of the patient for publication of this Case report and any accompanying images. A copy of the written consent is available for the review by the Editor-in-Chief of this journal.

## Competing interests

The authors declare that they have no competing interests.

## Authors’ contributions

MD carried out microarray and FISH testing, and drafted the manuscript. OH counseled the patient and critically reviewed the manuscript. All authors read and approved the final manuscript.
